# Supplementary material for: Network medicine for disease module identification and drug repurposing with the NeDRex platform
Source: Nat Commun. 2021 Nov 25;12:6848. doi: 10.1038/s41467-021-27138-2 (PMC8617287; doi:10.1038/s41467-021-27138-2)
Supplement: Supplementary file 1 — Supplementary Information [file 41467_2021_27138_MOESM1_ESM.pdf]

Supplementary Information

Network medicine for disease module identification and drug repurposing with the NeDRex platform

Sadegh *et al*.

Additional notes - Data integration and construction of NeDRexDB

NeDRexDB is a graph database that was constructed by integrating 10 source databases using a crowdsourcing framework. These 10 databases with their corresponding versions are shown in Supplementary Table 1.

Different databases store their data using various semantics, syntaxes, and potentially, in different locations. Consequently, writing parsers to extract data from multiple source databases is time consuming due to additional time investments of understanding the database schemas and vocabularies. To address this, we developed a crowdsourcing framework to develop database “descriptor” files. A JSON template file was developed with entries to describe each dataset, including version, upload date and column identifiers. Team members were assigned datasets and checked the licensing agreements, extracted the relevant data tables and filled in the JSON descriptor. The completed JSON descriptor and the downloaded data tables were stored in a folder together for parsing. A human-readable file was also filled in to describe the layout and contents of each data table.

Based on the descriptors, bespoke parsers were written for each dataset shown in Supplementary Table 1. These parsers extract entities (“nodes”) and the relationships between entities (“edges”), and store them in a MongoDB instance. MongoDB was chosen as the database for two primary reasons; firstly, MongoDB has a flexible schema, which provides the freedom to readily add new characteristics to documents in the database, whilst simultaneously allowing selective enforcement of certain guarantees. Secondly, MongoDB provides a rich set of operations for querying and updating, which facilitates data integration.

Note that the term “disorder” used in the metagraph and the NeDRexApp should be considered as the term “disease” in the paper.

Supplementary Table 1: Source databases integrated into NeDRexDB

Database	Date obtained (version, if known)	Nodes contributed	Edges contributed
OMIM[1]	2020-03-10*		Gene-[associated with]-Disorder
IID[2]	2020-02-11 (v2018-11)		Protein-[interacts with]-Protein
UniProt[3]	2020-02-11	Proteins	Gene-[encoded by]-Protein<br>Protein-[is isoform of]-Protein
Reactome[4]	2020-02-11	Pathways	Protein-[in pathway]-Pathway
DrugBank[5]	2020-02-11	Drugs	Drug-[has target]-Protein
DisGeNET[6] **	2019-12-02 (v6.0)		Gene-[associated with]-Disorder
DrugCentral[7]	2020-02-11 (v2018-08-26)		Drug-[has target]-Protein<br>Drug-[has indication]-Disorder<br>Drug-[has contraindication]-Disorder
Monarch Disease Ontology (MONDO)[8]	2020-02-11	Disorders	Disorder-[is subtype of]-Disorder
NCBI gene info[9]	2020-02-11	Genes	
InterPro[10]	2020-01-14 (DB: v77.0 & tool v5.40)	Signatures	Protein-[has signature]-Signature

* Updated weekly, but this date is the version used for the use cases in this paper.
** Only curated gene-disease associations from DisGeNET[6] are integrated.

Supplementary Table 2: Overview of different node and edge types in the NeDRexDB metagraph

Nodes	
Disorder	24,120
Drug	13,300
Gene	61,643
Pathway	2,309
Protein	212,745
Signature	36,025
**Edges**	
Disorder - [Is subtype of] - Disorder	38,210
Drug - [Has contraindication] - Disorder	12,591
Drug - [Has indication] - Disorder	3,906
Drug - [Has target] - Protein	29,932
Gene - [Associated with] - Disorder	33,378
Protein - [Is isoform of] - Protein	22,000
Drug - [Molecularly similar to] - Drug	168,866
Protein - [Encoded by] - Gene	33,162
Protein - [Has signature] - Signature	1,868,823
Protein - [In pathway] - Pathway	116,364
Protein - [Interacts with] - Protein	968,012
Protein - [Similar to] - Protein	10,831,760

Addition of edges to NeDRexDB from custom analyses

To add further edges to the database that could be exploited for module creation and drug-repurposing studies, three analyses were carried out to add edges to the database. Firstly, information about the signatures that proteins in the NeDRexDB had were identified by running InterPro Scan version 5.40 (dataset version 77.0). Secondly, protein similarity relationships were generated by calculating all-versus-all `blastp` (version 2.6.0+). Edges were added between two proteins if the E-value from `blastp` was lower than 1×10^{-3} in both directions (i.e., reciprocally). Finally edges were added between small molecule drugs based on molecular similarity. Molecular similarity was calculated using the Python RDKit library (version 2019.09.3). Implemented molecular similarity measures are Tanimoto similarity between 16,384-bit Morgan (circular) fingerprints at radii 1, 2, 3 and, 4, and Tanimoto similarity between Molecular Access System (MACCS) key fingerprints. This drug-drug similarity data, which is accessible via NeDRexAPI, can be especially interesting for users who want to employ their own drug repurposing methods utilizing machine learning or deep learning approaches.

After integration, the NeDRexDB comprises 350,142 nodes, distributed across six node types, and 14,127,004 edges, distributed across 12 edge types. The counts for these types are summarized in Supplementary Table 2. The metagraph of the relationship between nodes is shown in Supplementary Figure 1.

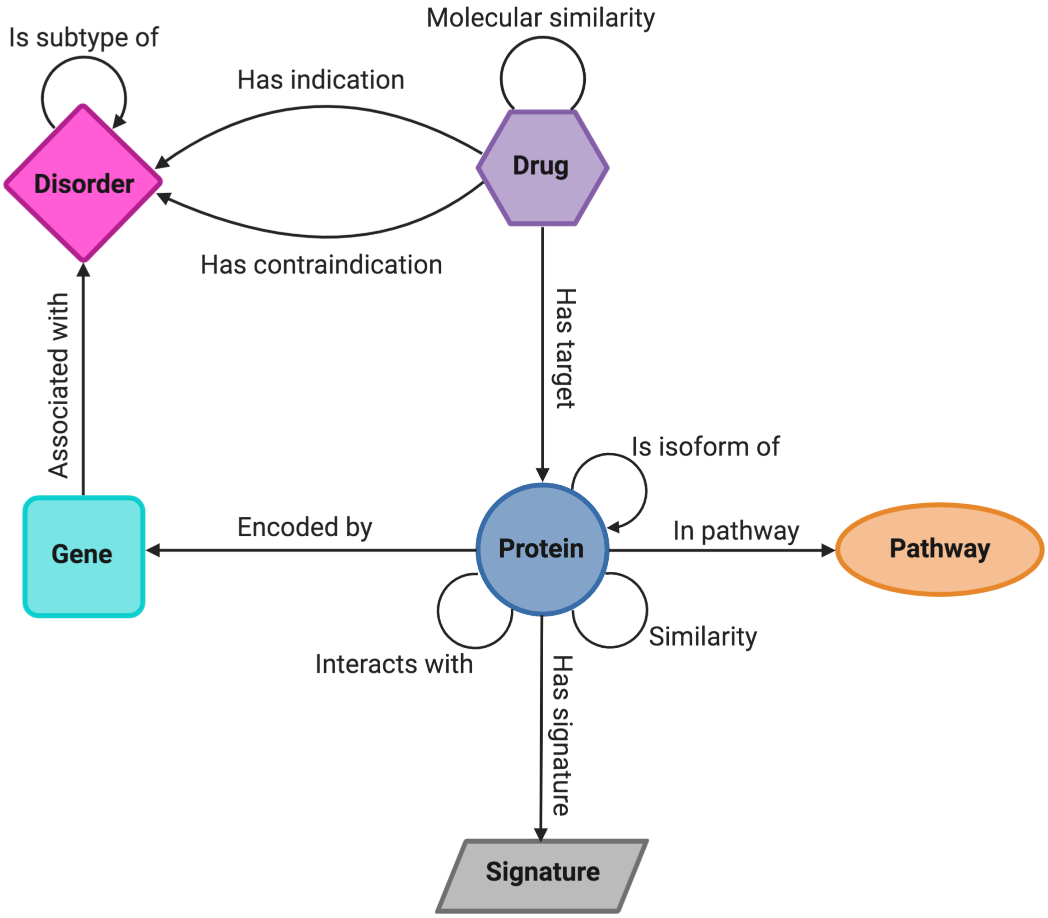

Supplementary Fig. 1 The NeDRexDB metagraph.

The metagraph illustrates all types of integrated relationships between different node types in NeDRexDB, some of which are used for algorithms in the NeDRex platform and can be imported into NeDRexApp. Note that while a number of the node and edge types are not used for the NeDRex algorithms and functions, users can access and download all of them via NeDRexAPI.

Guide for seed selection

For a disorder of interest, in case of using disease module identification algorithms, the seeds can be all or a subset of the genes associated with the disease. This set of “disease genes'' can be obtained from NeDRexDB using the NeDRexApp’s “Get Disease Genes” function. Users can select to include disease-gene associations from either OMIM, DisGeNET or union of both datasets. Alternatively, users can upload their own set of seeds, e.g. a list of differentially expressed genes (DEGs) or a hypothesis-driven selection of genes. In case of using drug ranking algorithms, the seeds can be the entire or part of the set of genes returned as the

disease module in the previous step. Alternatively, users can use disease genes or any custom list of genes directly as seeds for drug ranking tasks. Expert knowledge can be applied at all steps requiring the selection of seeds (Figure 1).

Our platform can also be used to identify disease modules and repurposable drugs for any newly discovered disease such as COVID-19. This could be implemented in multiple ways. For instance, users could select all or a subset of the SARS-CoV-2 interactors reported by Gordon et al.[11,12] as seeds to start the analysis using the methods available in NeDRex. Alternatively, DEGs from the differential gene expression analysis of COVID-19 patients from the study by Blanco-Melo et al.[13] could be used as seeds.

Supplementary Results

Algorithms parameters and seeds used for the use cases:

Using the NeDRexApp's "Get Disease Genes" function, the "Disease genes" for use cases are extracted based on the union of disease-gene associations integrated in NeDRexDB from OMIM and DisGeNET databases.

Use case 1: identification of disease pathways for ovarian cancer (OC), using MuST

Disease selection: mondo.0008170 (ovarian cancer), mondo.0006477 (undifferentiated ovarian carcinoma)

Disease genes: *AKT1, ALPK2, CDH1, CTNNB1, EPHB1, OPCML, PIK3CA, PRKN*

MuST:

Seeds	All disease genes for the selected disorders
Number of Steiner trees	5
Max number of iteration	5
Hub penalty	-

Use case 2: identification of therapeutic drugs for inflammatory bowel disease (IBD), using MuST and drug ranking algorithms

Disease selection: mondo.0005265 (inflammatory bowel disease)

Disease genes: *APC, APC2, ATG16L1, CARD9, CUL2, DEFA5, ICAM1, IL10, IL18RAP, IL23R, IL6, INAVA, IRGM, ITGA4, ITGAL, ITGB8, MUC19, NOD2, PLCG2, PTGS2, PTPN22, RASSF1, SFRP1, SFRP2, SLAMF8, SLC11A1, TGFB1, TNF, TNFSF15, VNN1*

MuST:

Seeds	All disease genes for IBD
Number of Steiner trees	5
Max number of iteration	5
Hub penalty	-

Closeness centrality:

Seeds	Genes from MuST disease module
Include only direct drugs	True
Include only approved drugs	True
Result size	50

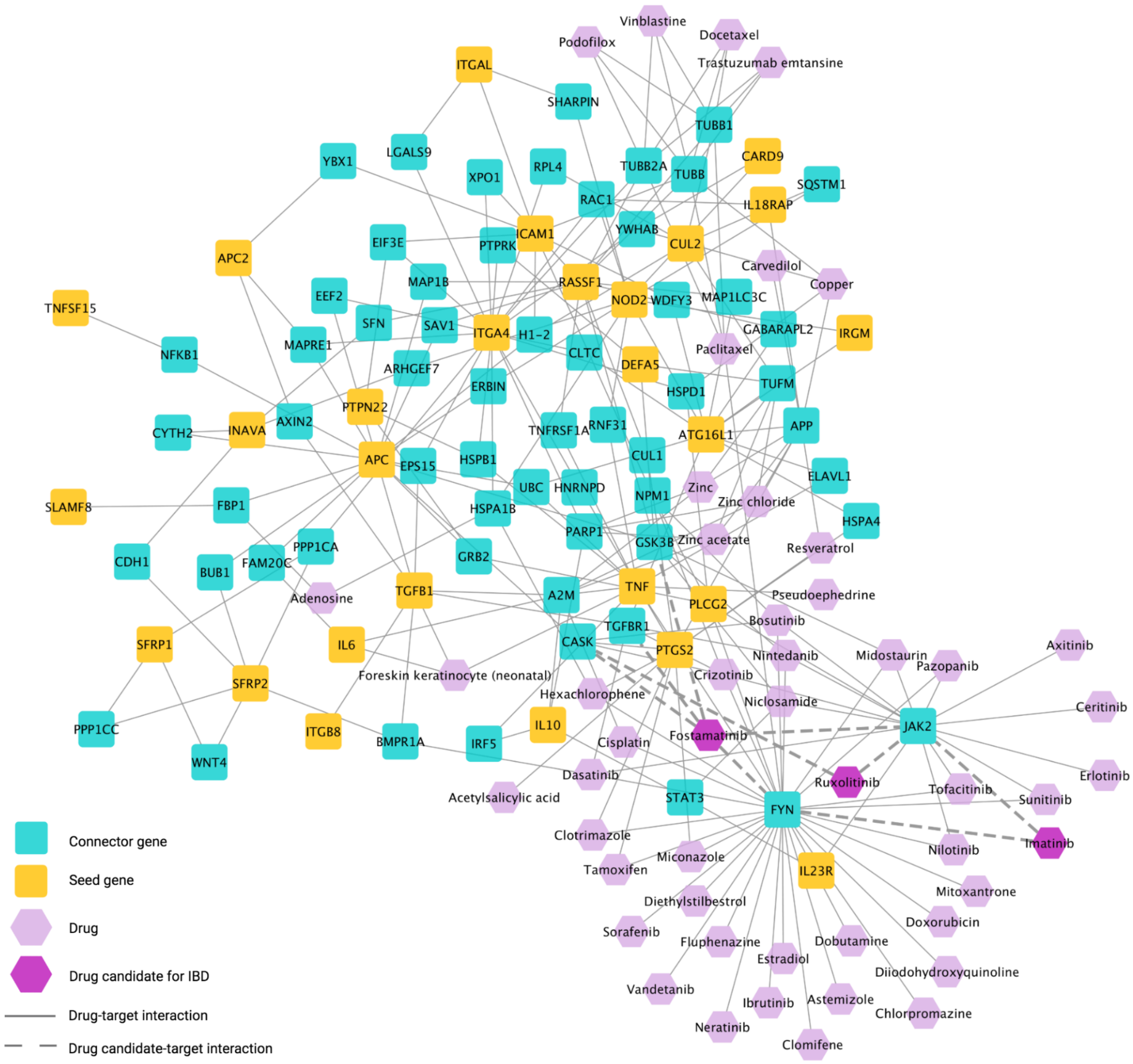

Supplementary Fig. 2 The inflammatory bowel disease module and its targeting top-ranked drugs. The IBD disease module derived by MuST method, combined with its targeting 25 top-ranked drugs by closeness centrality.

Use case 3: drug target and drug identification for pulmonary embolism (PE), using combination of DIAMOnD and TrustRank

Disease selection: mondo.0005279 (pulmonary embolism disease)

Disease genes: *CAT, DAB2IP, EPO, FGA, KLKB1, MERTK, MTHFR, PLAT, PLAU, TBXA2R, THBD,* and *XDH*

DIAMOnD:

Seeds	All disease genes for PE
Number of DIAMOnD genes	20
Weight of seeds	1
Return all edges	True

TrustRank:

Seeds	Genes from DIAMOnD disease module (excluding initial disease genes)
Include only direct drugs	True
Include only approved drugs	True
Damping factor	0.85
Result size	100

Use case 4: disease module and drug identification for Huntington's disease (HD), using BiCoN and TrustRank

Input: HD gene expression data (from GEO, accession number GSE3790). Patients with Vonsattel grades 2–4 and healthy controls.

BiCoN:

Input patient numerical data	HD gene expression data
Minimal solution subnetwork size	10
Maximal solution subnetwork size	15

TrustRank:

Seeds	All genes from BiCoN disease module
Include only direct drugs	True
Include only approved drugs	True

Damping factor	0.85
Result size	50

Use case 5: repurposed drugs for Alzheimer's disease (AD)

The disease genes for this use case are extracted based on the union of disease-gene associations integrated in NeDRexDB from OMIM and DisGeNET databases (applied score cutoff = 0.5).

A) Hypertension as original indication

Disease selection: mondo.0004975 (Alzheimer disease) and all its subtypes

Disease genes: *A2M, ABCA7, ACE, ADAM10, APOE, APP, BAX, BCL2, BDNF, CD2AP, CLU, GSK3B, HFE, IGF1R, IGF2, IL1B, INS, INSR, LEP, MPO, NOS3, NPY, PICALM, PLAU, PSEN1, PSEN2, SORL1, TREM2, AD5, AD6, AD7, AD8, AD10, AD11, AD12, AD13, AD14, AD15, AD16, AD17*

Closeness centrality:

Seeds	All disease genes for AD and its subtypes
Include only direct drugs	True
Include only approved drugs	True
Result size	100

B) Diabetes as original indication

Disease selection: mondo.0005015 (diabetes mellitus disease) and all its subtypes including

Disease genes: *ABCC8, ADIPOQ, AGPAT2, AKT2, ALMS1, APPL1, BLK, BSCL2, CAPN10, CAV1, CAVIN1, CCR5, CEL, CFTR, CTLA4, CTRC, DCAF17, DMXL2, DNAJC3, EIF2AK3, ENPP1, GATA6, GCGR, GCK, GLIS3, GPD2, HMGA1, HNF1A, HNF1B, HNF4A, IER3IP1, IGF2BP2, IL2RA, IL6, INS, INSR, IRS1, IRS2, ITPR3, KCNJ11, KLF11, LIPC, MAPK8IP1, MT-TL1, MTNR1B, NEUROD1, NSMCE2, PAX4, PDX1, PIK3R1, PLIN1, PPARG, PPP1R15B, PPP1R3A, PRSS1, PRSS2, PTF1A, PTPN1, PTPN22, RETN, SLC19A2, SLC29A3, SLC2A2, SLC30A8, SPINK1, SUMO4, TBC1D4, TCF7L2, TRMT10A, WFS1, XRCC4, ZFP57,* and genes

with entrez ids: 100188782, 100271697, 100303715, 3402, 3403, 3405, 3406, 3407, 3410, 3412, 3414, 4813, 50982, 57044, 8245, 8691

Disease selection: mondo.0004975 (Alzheimer disease) and all its subtypes

Disease genes: *A2M, ABCA7, ACE, ADAM10, APOE, APP, BAX, BCL2, BDNF, CD2AP, CLU, GSK3B, HFE, IGF1R, IGF2, IL1B, INS, INSR, LEP, MPO, NOS3, NPY, PICALM, PLAU, PSEN1, PSEN2, SORL1, TREM2, AD5, AD6, AD7, AD8, AD10, AD11, AD12, AD13, AD14, AD15, AD16, AD17*

No algorithm was used. The shared genes between the two disease gene sets: INS and INSR (*P*-value=0.017071)

C) Hyperlipidemia as original indication

Disease selection: mondo.0021187 (hyperlipidemia disease) and all its subtypes

Disease genes: *APOA2, APOA5, APOB, APOC2, APOE, CETP, EPHX2, GHR, GPIHBP1, LDLR, LDLRAP1, LIPC, LMF1, LPL, NOS3, PCSK9, PPP1R17, USF1, HYPLIP2*

DIAMOnD:

Seeds	All disease genes for Hyperlipidemia and its subtypes
Number of DIAMOnD genes	200
Weight of seeds	1
Return all edges	False

Disease selection: mondo.0004975 (Alzheimer disease) and all its subtypes

Disease genes: *A2M, ABCA7, ACE, ADAM10, APOE, APP, BAX, BCL2, BDNF, CD2AP, CLU, GSK3B, HFE, IGF1R, IGF2, IL1B, INS, INSR, LEP, MPO, NOS3, NPY, PICALM, PLAU, PSEN1, PSEN2, SORL1, TREM2, AD5, AD6, AD7, AD8, AD10, AD11, AD12, AD13, AD14, AD15, AD16, AD17*

DIAMOnD:

Seeds	All disease genes for AD and its subtypes
Number of DIAMOnD genes	200
Weight of seeds	1
Return all edges	False

The shared genes between the disease modules of AD and hyperlipidemia: *A2M, APOE, APP, CLU, IGF2, NOS3,* and *PLAU* (*P*-value=0.023827)

Closeness centrality:

Seeds	Shared genes between AD and hyperlipidemia disease modules
Include only direct drugs	False
Include only approved drugs	True
Result size	50

Alternatively, Gemfibrozil can also be retrieved by prioritizing the drugs targeting directly the AD disease module with TrustRank function (rank 62), given that the direct targets of this drug belong to the AD-module. The latter denotes that there is not only one approach to retrieve repurposable drugs; using the indirect and direct modes, Gembrozil appears as a potential repurposable drug.

TrustRank:

Seeds	All genes from AD DIAMOnD disease module
Include only direct drugs	True
Include only approved drugs	True
Damping factor	0.85
Result size	200

Statistical validation of results for the use cases:

For evaluation of results returned by NeDRex for the drug repurposing use cases, a list of drugs as the true reference list was compiled. This reference list contains indicated drugs for the treatment of each use case disease, which can be obtained directly from NeDRexDB or other resources. Since drug indication data from DrugBank is not available via non-commercial license and was therefore not integrated into the open access NeDRexDB. The list of indicated drugs was complemented by browsing DrugBank directly. For the use cases where we could only retrieve a few indicated drugs (less than 10), the reference list was extended by drugs from clinical trials or supported by literature evidence (with at least five references as evidence) from CTD database[14]. The reference lists of drugs with the empirical *P*-values for each use case are reported in the following. The reported *P*-values are rounded to three significant digits and values smaller than 0.001 were indicated correspondingly. The drug candidates discussed in the main paper are not necessarily all among the reference list of drugs. The idea behind some use cases was to explore the predicted drugs beyond the already known therapeutic options.

Use case: OC

**Validation type**		**CTD with >= 5 references + DrugBank indicated for OC**
**Disease module**	Empirical *P*-value	0.044
	Empirical *P*-value (precision-based)	0.255

Validation function settings:1000 permutations, all drugs

Drugs from DrugBank source indicated for the disease:

DB00445	DB00290	DB01229
DB01181	DB00773	DB00762
DB00970	DB00675	DB00642

Drugs from CTD database with more than 5 references for the drug-disease association (only those that could be mapped to a DrugBank ID and hence exist in the NeDRexDB):

DB00158	DB06732	DB00563	DB04216	DB03733	DB00313
DB00624	DB00602	DB00396	DB11132	DB09536	DB00853

DB00550	DB00675	DB03843	DB11841	DB00316	DB04539
DB01174	DB01229	DB00255	DB00898	DB01645	DB11091
DB01234	DB00755	DB01262	DB00122	DB14085	DB01169
DB09325	DB09526	DB00134	DB06767	DB00997	DB00136
DB12116	DB00783	DB05076	DB00636	DB00907	DB00412
DB00435	DB00182	DB00531	DB00197	DB00515	DB00564

Use case: IBD

Validation type		CTD with >= 10 references + DrugBank indicated for IBD
**Drug list**	Empirical *P*-value	<0.001
	DCG-based empirical *P*-value	<0.001
**Disease module**	Empirical *P*-value	0.017
	Empirical *P*-value (precision-based)	0.036
**Joint module & drug**	Empirical *P*-value	0.777
	Empirical *P*-value (precision-based)	<0.001

Validation function settings:10000 permutations, all drugs

Drugs from DrugBank source indicated for the disease: DB00836, DB13248

Drugs from CTD database with more than 10 references for the drug-disease association (only those that could be mapped to a DrugBank ID and hence exist in the NeDRexDB):

DB09140	DB00927	DB01234	DB00403	DB04348	DB00763
DB00437	DB07715	DB09130	DB11588	DB00512	DB01222
DB12025	DB00761	DB00533	DB13242	DB09061	DB02709
DB00608	DB04557	DB13721	DB00515	DB00806	DB01094
DB00907	DB01238	DB12116	DB01136	DB02587	DB00669
DB01039	DB12965	DB06732	DB00853	DB00177	DB04930
DB00995	DB00834	DB11136	DB04743	DB12243	DB01698
DB00843	DB14512	DB13063	DB13323	DB11525	DB05076
DB00544	DB01586	DB07352	DB01593	DB09201	DB04398

DB00947	DB00997	DB00471	DB00143	DB01645	DB00531
DB00675	DB01041	DB00783	DB00746	DB01159	DB00338
DB11135	DB01956	DB00563	DB01009	DB00877	DB07767
DB00762	DB00916	DB00255	DB00678	DB08839	DB11841
DB00755	DB00201	DB11695	DB11874	DB08818	DB00197
DB01216	DB02736	DB06774	DB04221	DB00523	DB06777
DB00982	DB00586	DB00641	DB00781	DB06151	DB12881
DB00396	DB03166	DB00537	DB00951	DB01030	DB00715
DB03843	DB13182	DB00317	DB00435	DB01042	DB00566
DB04173	DB00428	DB01132	DB01919	DB11091	DB09536
DB00744	DB00398	DB09321	DB00482	DB00126	DB00290
DB03796	DB01016	DB11132	DB00441	DB00412	DB00470
DB00162	DB00795	DB12870	DB00811	DB00859	DB08059
DB14154	DB14184	DB01592	DB09526	DB04115	DB01097
DB01050	DB14183	DB08604	DB00182	DB00966	DB12622
DB00169	DB00295	DB01093	DB14180	DB00158	DB03619
DB00640	DB13765	DB00636	DB08895	DB00518	DB00331
DB00724	DB03518	DB00122	DB01262	DB00603	DB00499
DB01118	DB01296	DB00184	DB00134	DB01174	DB01221
DB05381	DB12695	DB00277	DB04573	DB00399	DB00313
DB00502	DB00720	DB00888	DB11342	DB00788	DB00970
DB04216	DB08398	DB00584	DB05767	DB04819	DB00635
DB06155	DB00136	DB06536	DB00495	DB14066	DB09086
DB00388	DB06530	DB00661	DB00262	DB09325	DB11672
DB01268	DB02994	DB07374	DB00163	DB00759	DB07795
DB06803	DB00421	DB00316	DB11231	DB01024	DB11109
DB06767	DB02546	DB09322	DB00863	DB00959	DB01169
DB00605	DB11457	DB08162	DB13751	DB04827	DB04539
DB01120	DB00682	DB00860	DB13318	DB00602	DB01149
DB01611	DB01954	DB13172	DB01085	DB01229	DB14533
DB00993	DB06510	DB00917	DB00503	DB00188	DB12422
DB12510	DB00624	DB14085	DB11831	DB00864	DB00564
DB00928	DB00550	DB00526	DB00898	DB00554	DB01241

Use case: PE

a) Excluding seeds: (following the exact scenario from the paper)

Validation type		NeDRexDB + DrugBank indicated for PE
**Drug list**	Empirical *P*-value	<0.001
	DCG-based empirical *P*-value	<0.001
**Disease module**	Empirical *P*-value	<0.001
	Empirical *P*-value (precision-based)	0.007
**Joint module & drug**	Empirical *P*-value	<0.001
	Empirical *P*-value (precision-based)	0.018

b) Entire disease module including seeds:

Validation type		NeDRexDB + DrugBank indicated for PE
**Drug list**	Empirical *P*-value	<0.001
	DCG-based empirical *P*-value	<0.001
**Disease module**	Empirical *P*-value	<0.001
	Empirical *P*-value (precision-based)	0.012
**Joint module & drug**	Empirical *P*-value	<0.001
	Empirical *P*-value (precision-based)	0.010

Validation function settings:1000 permutations, only approved drugs

Drugs from DrugBank source indicated for the disease:

DB00569	DB01109	DB06228
DB09255	DB00682	DB09075
DB06605	DB00320	DB00013
DB13327	DB01418	DB06695

DB08813	DB00086	

Drugs from NeDRexDB source indicated for the disease*:

DB00013	DB09075	DB09258
DB00009	DB01418	

* Used the disease node mondo.0005279 to obtain drugs indicated in the disease

Use case: HD

**Validation type**		**DrugBank indicated + clinical trials for HD**
**Drug list**	Empirical *P*-value	<0.001
	DCG-based empirical *P*-value	0.011
**Disease module**	Empirical *P*-value	0.003
	Empirical *P*-value (precision-based)	0.180
**Joint module & drug**	Empirical *P*-value	0.048
	Empirical *P*-value (precision-based)	0.048

Validation function settings:1000 permutations, only approved drugs

Drugs from DrugBank source indicated for the disease:

DB12161	DB00623	DB04844
DB00915	DB13025	DB00502

Drugs from DrugBank source in clinical trials for the disease:

DB00470	DB00289	DB00121	DB01156	DB01235	DB01954
DB07138	DB01043	DB00334	DB13004	DB00682	DB09061
DB09321	DB00494	DB00740	DB00502	DB13025	DB14509
DB08887	DB00313	DB01039	DB00148	DB00338	DB06819
DB00908	DB11677	DB11915	DB01104	DB00152	DB09535
DB13134	DB06685	DB00514	DB08387	DB15155	DB00268
DB12161	DB04844	DB09081	DB00390	DB04868	DB00734

DB00331	DB11725	DB00915	DB01017	DB01026	DB09341
DB00413	DB09270	DB11947	DB00215	DB11062	DB01586
DB12542	DB00980	DB00323	DB00190	DB00404	DB01954

Use case: AD

Notice that for the Alzheimer's disease use case, the NeDRex standard pipeline for drug repurposing is not used. This use case is hypothesis-driven and we aim to extract possibly repurposable drugs which are indicated for diseases that are known to be associated with AD. For this purpose, we rely on an exploratory approach and the *P*-values for this approach are reported in the following:

A) Hypertension as original indication

**Validation type**		**DrugBank indicated + clinical trials for AD**
**Drug list**	Empirical *P*-value	<0.001
	DCG-based empirical *P*-value	<0.001

Validation function settings:1000 permutations, only approved drugs

B) Diabetes as original indication

**Validation type**		**DrugBank indicated + clinical trials for AD**
**Drug list**	Empirical *P*-value	0.002

Validation function settings:1000 permutations, all drugs

All drugs targeting two genes of INS and INSR (shared genes between AD and diabetes associated genes) are considered as the list of drugs to be validated.

C) Hyperlipidemia as original indication

**Validation type**		**DrugBank indicated + clinical trials for AD**
**Drug list**	Empirical *P*-value	<0.001

	DCG-based empirical *P*-value	<0.001
**Disease module**	Empirical *P*-value	0.037
	Empirical *P*-value (precision-based)	0.079

Validation function settings:1000 permutations, only approved drugs

Seven overlapping genes mentioned in the paper are considered as the module for validation. The drugs returned by the closeness centrality method for this module are considered as the list of drugs to be validated.

Drugs from DrugBank source indicated for the disease:

DB00656	DB12274	DB00674	DB01043	DB16599
DB00334	DB09081	DB00679	DB00457	DB00843

Drugs from DrugBank source in clinical trials for the disease:

DB00983	DB00099	DB00175	DB00683	DB16599	DB11867
DB00165	DB15161	DB12129	DB00850	DB12635	DB01273
DB16205	DB01576	DB12463	DB05708	DB00158	DB13065
DB05881	DB11094	DB11957	DB00973	DB00759	DB00005
DB00186	DB11715	DB09153	DB00201	DB04660	DB00482
DB12132	DB00966	DB08860	DB00481	DB11664	DB12274
DB12145	DB00603	DB16541	DB00370	DB00674	DB01156
DB06280	DB00788	DB09210	DB00813	DB12540	DB15120
DB01438	DB00125	DB01914	DB00166	DB01224	DB12680
DB11726	DB06138	DB00218	DB02546	DB05271	DB12034
DB00030	DB00334	DB00285	DB00682	DB08929	DB08834
DB00196	DB09331	DB04926	DB15307	DB00360	DB00215
DB12620	DB04864	DB05586	DB15079	DB00254	DB00425
DB14814	DB16213	DB00020	DB11756	DB05458	DB06712
DB01306	DB00601	DB01065	DB00413	DB00313	DB12790
DB01238	DB00372	DB00126	DB05938	DB00028	DB00422
DB00390	DB16274	DB15033	DB06292	DB05832	DB00747
DB11887	DB06393	DB11062	DB00459	DB12621	DB01381

DB12285	DB11135	DB13082	DB00563	DB12368	DB00328
DB00062	DB11859	DB15317	DB01399	DB00996	DB08839
DB00060	DB00171	DB05150	DB00556	DB01404	DB12551
DB12201	DB00982	DB09420	DB01175	DB12288	DB15130
DB11526	DB01104	DB01220	DB12819	DB00307	DB11959
DB00533	DB00396	DB08824	DB00641	DB01003	DB11893
DB00471	DB00577	DB16044	DB01247	DB15234	DB05119
DB09241	DB00457	DB00734	DB00740	DB01367	DB07138
DB00783	DB01050	DB00864	DB11953	DB04216	DB12969
DB00989	DB09150	DB02709	DB06527	DB01045	DB14914
DB01275	DB00502	DB04942	DB00316	DB00699	DB12747
DB01076	DB12263	DB06156	DB14945	DB00818	DB00514
DB01132	DB04540	DB15192	DB05596	DB14329	DB01276
DB00358	DB11805	DB01235	DB12689	DB00834	DB13961
DB12583	DB01041	DB00338	DB00572	DB09535	DB11752
DB09148	DB14790	DB00956	DB01656	DB01309	DB12702
DB00184	DB01043	DB06077	DB05898	DB00624	DB09034
DB01307	DB00421	DB00133	DB05450	DB12310	DB14580
DB00289	DB00633	DB15729	DB01254	DB12897	DB06819
DB05010	DB15391	DB15528	DB08887	DB12176	DB00790
DB15763	DB14476	DB00331	DB13453	DB06168	DB05679
DB12504	DB12116	DB12287	DB06090	DB02701	DB00470
DB16647	DB00007	DB11674	DB00656	DB08872	DB11721
DB00745	DB12501	DB00635	DB06622	DB01638	DB12547
DB01018	DB06247	DB09151	DB03255	DB09321	DB01236
DB00163	DB05454	DB09149	DB11931	DB11725	DB01022
DB12717	DB00404	DB11910	DB05592	DB01167	DB11748
DB00297	DB16674	DB00843	DB01026	DB14322	DB15376
DB14491	DB16297	DB12057	DB06725	DB00908	DB11672
DB16344	DB00486	DB06267	DB15058	DB05400	DB01017
DB00150	DB00046	DB00169	DB09061	DB09128	DB04868
DB15135	DB05289	DB01202	DB00178	DB01278	DB15155
DB14509	DB00753	DB14933	DB03756	DB01136	DB12229
DB00877	DB08842	DB06140	DB05308	DB06655	
DB11133	DB00115	DB09422	DB00412	DB13134	

Supplementary References

1. Amberger, J. S., Bocchini, C. A., Scott, A. F. & Hamosh, A. OMIM.org: leveraging knowledge across phenotype–gene relationships. *Nucleic Acids Res.* **47**, D1038–D1043 (2018).
2. Kotlyar, M., Pastrello, C., Malik, Z. & Jurisica, I. IID 2018 update: context-specific physical protein–protein interactions in human, model organisms and domesticated species. *Nucleic Acids Res.* **47**, D581–D589 (2018).
3. The UniProt Consortium. UniProt: a worldwide hub of protein knowledge. *Nucleic Acids Res.* **47**, D506–D515 (2018).
4. Jassal, B. *et al.* The reactome pathway knowledgebase. *Nucleic Acids Res.* **48**, D498–D503 (2019).
5. Wishart, D. S. *et al.* DrugBank 5.0: a major update to the DrugBank database for 2018. *Nucleic Acids Res.* **46**, D1074–D1082 (2017).
6. Piñero, J. *et al.* The DisGeNET knowledge platform for disease genomics: 2019 update. *Nucleic Acids Res.* **48**, D845–D855 (2019).
7. Ursu, O. *et al.* DrugCentral 2018: an update. *Nucleic Acids Res.* **47**, D963–D970 (2018).
8. Mungall, C. J. *et al.* The Monarch Initiative: an integrative data and analytic platform connecting phenotypes to genotypes across species. *Nucleic Acids Res.* **45**, D712–D722 (2016).
9. Maglott, D., Ostell, J., Pruitt, K. D. & Tatusova, T. Entrez Gene: gene-centered information at NCBI. *Nucleic Acids Res.* **39**, D52–D57 (2010).
10. Blum, M. *et al.* The InterPro protein families and domains database: 20 years on. *Nucleic Acids Res.* **49**, D344–D354 (2020).
11. Gordon, D. E. *et al.* A SARS-CoV-2 protein interaction map reveals targets for drug

repurposing. *Nature* **583**, 459–468 (2020).

12. Gordon, D. E. *et al.* Comparative host-coronavirus protein interaction networks reveal pan-viral disease mechanisms. *Science* **370**, (2020).
13. Blanco-Melo, D., Nilsson-Payant, B., Liu, W. C. & Møller, R. SARS-CoV-2 launches a unique transcriptional signature from in vitro, ex vivo, and in vivo systems. *BioRxiv* (2020).
14. Davis, A. P. *et al.* Comparative Toxicogenomics Database (CTD): update 2021. *Nucleic Acids Res.* **49**, D1138–D1143 (2021).
