# Supplementary material for: Network medicine for disease module identification and drug repurposing with the NeDRex platform
Source: Nat Commun. 2021 Nov 25;12:6848. doi: 10.1038/s41467-021-27138-2 (PMC8617287; doi:10.1038/s41467-021-27138-2)
Supplement: Supplementary file 3 — Reporting Summary [file 41467_2021_27138_MOESM3_ESM.pdf]

nature portfolio

Corresponding author(s): Sepideh Sadegh
Last updated by author(s): Nov 3, 2021

Reporting Summary

Nature Portfolio wishes to improve the reproducibility of the work that we publish. This form provides structure for consistency and transparency in reporting. For further information on Nature Portfolio policies, see our Editorial Policies and the Editorial Policy Checklist.

Statistics

For all statistical analyses, confirm that the following items are present in the figure legend, table legend, main text, or Methods section.

n/a	Confirmed	
☒	☐	The exact sample size (*n*) for each experimental group/condition, given as a discrete number and unit of measurement
☒	☐	A statement on whether measurements were taken from distinct samples or whether the same sample was measured repeatedly
☐	☒	The statistical test(s) used AND whether they are one- or two-sided<br>*Only common tests should be described solely by name; describe more complex techniques in the Methods section.*
☒	☐	A description of all covariates tested
☒	☐	A description of any assumptions or corrections, such as tests of normality and adjustment for multiple comparisons
☒	☐	A full description of the statistical parameters including central tendency (e.g. means) or other basic estimates (e.g. regression coefficient) AND variation (e.g. standard deviation) or associated estimates of uncertainty (e.g. confidence intervals)
☐	☒	For null hypothesis testing, the test statistic (e.g. *F*, *t*, *r*) with confidence intervals, effect sizes, degrees of freedom and *P* value noted<br>*Give P values as exact values whenever suitable.*
☒	☐	For Bayesian analysis, information on the choice of priors and Markov chain Monte Carlo settings
☒	☐	For hierarchical and complex designs, identification of the appropriate level for tests and full reporting of outcomes
☒	☐	Estimates of effect sizes (e.g. Cohen's *d*, Pearson's *r*), indicating how they were calculated

Our web collection on statistics for biologists contains articles on many of the points above.

Software and code

Policy information about availability of computer code

Data collection	All the data supporting the findings of this study are available publicly and their integration is described accordingly within the paper and its supplementary information files. The NeDRexDB source code is publicly available under the GPL3 license in the Github repository: https://github.com/repotrial/nedrex<br>The following packages and software are used for database construction, API implementation, and task processing: BLAST (v2.9.0+), Neo4j (v3.5.12 community), MongoDB (v4.0.10). In addition, Python (v3.7.7) was used with the following libraries: biopython (v1.77), docker (v4.3.1), fastapi (v0.61.0), mongoengine (v0.19.1), neo4j (v4.1.1), networkx (v2.4), numpy (v1.19.5), pandas (v1.3.3), pymongo (v3.10.1).
Data analysis	The NeDRex code is openly available on GitHub repositories (https://github.com/repotrial/nedrex and https://github.com/repotrial/NeDRexApp) under the terms of the GNU General Public License, Version 3.<br>The following packages and softwares are used for the implementation of the network-based algorithms and validation methods: DIAMOnD was obtained from https://github.com/dinaghiassian/DIAMOnD, using the 22nd Sept 2020 commit, bicon (version 1.2.11), graph-tool library (v. 2.35), pandas (v1.3.3), networkx (v2.2), MuST (v 1.0), jgrapht 1.4.0. NeDRexApp (v. 1.1.1) for Cytoscape 3 is written in Java (JDK 8).

For manuscripts utilizing custom algorithms or software that are central to the research but not yet described in published literature, software must be made available to editors and reviewers. We strongly encourage code deposition in a community repository (e.g. GitHub). See the Nature Portfolio guidelines for submitting code & software for further information.

Data

Policy information about availability of data

All manuscripts must include a data availability statement. This statement should provide the following information, where applicable:

- Accession codes, unique identifiers, or web links for publicly available datasets
- A description of any restrictions on data availability
- For clinical datasets or third party data, please ensure that the statement adheres to our policy

The authors declare that the NeDRexDB knowledgebase supporting the findings of this study are available via https://api.nedrex.net/. The construction of NeDRexDB is described accordingly within the paper and its Supplementary Information files. The NeDRexDB knowledgebase contains information obtained from the Online Mendelian Inheritance in Man® (OMIM®) database, which has been obtained through a license from the Johns Hopkins University, which owns the copyright thereto. Use of the NeDRex dataset is governed by an End User License Agreement (available at https://nedrex.net/about.html), due to requirements of including OMIM as a source database.
The following databases are used in this study: IID (http://iid.ophid.utoronto.ca/), DrugBank (https://go.drugbank.com/), DrugCentral (https://drugcentral.org/), DisGeNET (https://www.disgenet.org/), OMIM (https://omim.org/), NCBI gene info (https://www.ncbi.nlm.nih.gov/gene), UniProt (https://www.uniprot.org/), MONDO (https://mondo.monarchinitiative.org/) and Reactome (https://reactome.org/).

Field-specific reporting

Please select the one below that is the best fit for your research. If you are not sure, read the appropriate sections before making your selection.

- [x] Life sciences
- [ ] Behavioural & social sciences
- [ ] Ecological, evolutionary & environmental sciences

For a reference copy of the document with all sections, see nature.com/documents/nr-reporting-summary-flat.pdf

Life sciences study design

All studies must disclose on these points even when the disclosure is negative.

Sample size	n/a - As stated in the manuscript, we did not carry out any clinical experiments and none of the datasets integrated in the NeDRex contain patient-level data.
Data exclusions	During integration step to build NeDRexDB, no data from the original datasets were excluded.
Replication	We ran each use cases 10 times and always obtained the same results. However, none of the implemented algorithms in NeDRex is fully deterministic, therefore, slight changes in the results might occur in running the same analyses pipeline several times. Also future updates on the databases can affect the reproducibility.
Randomization	For each use case we computed empirical P-values based on the background models generated from 1000 randomizations.
Blinding	n/a - As stated in the manuscript, we did not carry out any clinical experiments.

Reporting for specific materials, systems and methods

We require information from authors about some types of materials, experimental systems and methods used in many studies. Here, indicate whether each material, system or method listed is relevant to your study. If you are not sure if a list item applies to your research, read the appropriate section before selecting a response.

Materials & experimental systems

n/a	Involved in the study	
[x]	[ ]	Antibodies
[x]	[ ]	Eukaryotic cell lines
[x]	[ ]	Palaeontology and archaeology
[x]	[ ]	Animals and other organisms
[x]	[ ]	Human research participants
[x]	[ ]	Clinical data
[x]	[ ]	Dual use research of concern

Methods

n/a	Involved in the study	
[x]	[ ]	ChIP-seq
[x]	[ ]	Flow cytometry
[x]	[ ]	MRI-based neuroimaging
